# Accurate MR Image Registration to Anatomical Reference Space for Diffuse Glioma

**DOI:** 10.3389/fnins.2020.00585

**Published:** 2020-06-05

**Authors:** Martin Visser, Jan Petr, Domenique M. J. Müller, Roelant S. Eijgelaar, Eef J. Hendriks, Marnix Witte, Frederik Barkhof, Marcel van Herk, Henk J. M. M. Mutsaerts, Hugo Vrenken, Jan C. de Munck, Philip C. De Witt Hamer

**Affiliations:** ^1^Department of Radiology and Nuclear Medicine, Amsterdam UMC, Amsterdam, Netherlands; ^2^Institute of Radiopharmaceutical Cancer Research, Helmholtz-Zentrum Dresden-Rossendorf, Dresden, Germany; ^3^Cancer Center Amsterdam, Brain Tumor Center, Department of Neurosurgery, Amsterdam UMC, Amsterdam, Netherlands; ^4^Department of Radiotherapy, Netherlands Cancer Institute, Amsterdam, Netherlands; ^5^UCL Queen Square Institute of Neurology, University College London, London, United Kingdom; ^6^UCL Institute of Healthcare Engineering, University College London, London, United Kingdom; ^7^Division of Cancer Sciences, Manchester Cancer Research Centre, School of Medical Sciences, Faculty of Biology, Medicine and Health, Manchester Academic Health Science Centre, The University of Manchester, Manchester, United Kingdom

**Keywords:** glioma, magnetic resonance imaging, image processing, computer-assisted, linear registration, non-linear registration

## Abstract

To summarize the distribution of glioma location within a patient population, registration of individual MR images to anatomical reference space is required. In this study, we quantified the accuracy of MR image registration to anatomical reference space with linear and non-linear transformations using estimated tumor targets of glioblastoma and lower-grade glioma, and anatomical landmarks at pre- and post-operative time-points using six commonly used registration packages (FSL, SPM5, DARTEL, ANTs, Elastix, and NiftyReg). Routine clinical pre- and post-operative, post-contrast T1-weighted images of 20 patients with glioblastoma and 20 with lower-grade glioma were collected. The 2009a Montreal Neurological Institute brain template was used as anatomical reference space. Tumors were manually segmented in the patient space and corresponding healthy tissue was delineated as a target volume in the anatomical reference space. Accuracy of the tumor alignment was quantified using the Dice score and the Hausdorff distance. To measure the accuracy of general brain alignment, anatomical landmarks were placed in patient and in anatomical reference space, and the landmark distance after registration was quantified. Lower-grade gliomas were registered more accurately than glioblastoma. Registration accuracy for pre- and post-operative MR images did not differ. SPM5 and DARTEL registered tumors most accurate, and FSL least accurate. Non-linear transformations resulted in more accurate general brain alignment than linear transformations, but tumor alignment was similar between linear and non-linear transformation. We conclude that linear transformation suffices to summarize glioma locations in anatomical reference space.

## Introduction

Tumor location is important when comparing treatment and outcome between populations of patients with diffuse glioma. The distribution of tumor locations can be summarized and compared across patients by transforming tumor segmentations from the individual patient space to a common anatomical reference space. Tumor location, biopsy decision, and residual tumor after surgery can then be summarized into tumor, biopsy, or resection probability maps, respectively. These summaries can provide answers to research questions on glioma-genesis (Steed et al., 2016), location preference for molecular sub-types (Ellingson et al., 2012, 2013), differences in surgical intervention (De Witt Hamer et al., 2013; Müller et al., 2019), survival prediction (Liu et al., 2016), and location of neuropsychological domains (Hendriks et al., 2018).

Patient images are brought into spatial alignment with the anatomical reference space through image registration. There is a large number of different approaches to image registration, which differ in the registration paradigm (e.g., the degrees of freedom), similarity metrics, regularization, and the choices in optimization (Viergever et al., 2016). Due to the diversity in implementation of these characteristics, various (publicly available) registration packages provide different solutions to the same problem.

The accuracy of inter-subject brain MRI registration was studied for several public software packages with publicly available data of eighty healthy individuals, showing the added value of high-degree-of freedom (non-linear) registrations (Klein et al., 2009). However, registration becomes more challenging when transforming images of brain tumors, lesions, atrophy, or deformed brain to an anatomical reference space (Crinion et al., 2007; Ripollés et al., 2012). For post-operative recurrent brain tumor patients specifically, this was studied in a group of eight patients by Ou et al. (2014).

Glioma registration has two main challenges. First, the normal tissue contrast is altered by the tumor components and surrounding edema. And second, mass effect from the tumor and from surgery alters the volume and shape of normal tissue. These challenges potentially diminish the registration accuracy, resulting in less reliable summary maps.

The determination of image registration accuracy is not standardized, nor well defined. Previous work determined accuracy of different registration algorithms by comparing difference between extrinsic (i.e., bone-implanted fiducials) with intrinsic (i.e., intensity values in the images) registration (West et al., 1997). Currently intrinsic registration has become dominant in literature (Viergever et al., 2016), where the ground truth of patient attached fiducials is lacking. Most commonly registration results are checked visually, a qualitative approach which is rater dependent. Manually segmenting structures in the images allows for quantitative comparison (Caviness et al., 1996; Shattuck et al., 2008), but is time-consuming and subject to rater variability. Another quantitative method is placing landmarks in the images (Chollet et al., 2014). Accuracy is most reliably measured when using several forms of manual annotation combined (Rohlfing, 2012).

In this study we compared the registration accuracy to anatomical reference space for tumor alignment and for anatomical landmark alignment of 20 patients diagnosed with glioblastoma and 20 with lower-grade glioma, between pre- and post-operative MRI time-points, between six publicly available registration packages, and between linear and non-linear registrations.

## Materials and Methods

### Patients

Patients were randomly selected from a cohort treated at the Neurosurgical Center of Amsterdam UMC, location VUmc, between 2009 and 2013, and were previously reported on in an inter-rater agreement study (Visser et al., 2019). The MR data of 40 glioma patients was used, consisting of 20 patients with histopathologically confirmed glioblastoma and 20 patients with lower-grade glioma.

### Imaging and Anatomical Reference Space

Imaging was performed on a variety of systems (Siemens, model Sonata or Avanto; GE medical systems, model Signa HDxt or DISCOVERY MR750; Toshiba, model Titan3T; Philips, model Panorama HFO or Ingenuity) with a field strength of 1T (1% of all scans), 1.5T (62% of all scans), or 3T (37% of all scans). The standardized protocol included sagittal 3D turbo fluid-attenuated inversion-recovery (FLAIR) images [repetition time/echo time/inversion time (TR/TE/TI) 4,800–8,000/125–400/1,650–2,200 ms] with 1.3-mm slice thickness, axial T2-weighted turbo spin echo images (TR/TE 5,190–8,670/93–101 ms) with 5-mm slice thickness, and 3D T1-weighted MPRAGE (TR/TE/TI 2700/1.5/950 ms)/3-D FSGPR (TR/TE/TI 6.6/3/450 ms)/3D TFE (TR/TE 7/3 ms) images with 0.5–1/0.5–1/1–1.5 mm voxel size.

Following clinical protocol at the treatment site, the pre-operative MRI was made within 1 week before resection. The MRI after surgery was made within 72 h after resection for glioblastoma and about 4 months after resection for lower-grade glioma.

Only post-contrast T1-weighted MRI (T1c) images were used for registrations in this study, and were registered individually to the anatomical reference space, for which the symmetric Montreal Neurological Institute 09a (ICBM2009a space) atlas^1^ was used (Fonov et al., 2009, 2011).

### Manual Annotations

A single rater, a neurosurgeon with 20 years clinical experience, segmented the gliomas in MRI at both the pre- and post-operative time-point for all 40 patients. Segmentations in patient space were made with the semi-automatic SmartBrush tool (BrainLab, Feldkirchen, Germany). Radiological presentation of glioma can be divided into MR non-enhancing and contrast-enhancing tumors. Although exceptions exist, many non-enhancing gliomas are histopathologically diagnosed as lower-grade glioma (WHO grades II or III), and many contrast-enhancing gliomas as glioblastoma (Scott et al., 2002). For glioblastoma, the tumor segmentation was defined as enhancing tumor elements including enclosed necrosis. The lower-grade gliomas were segmented on T2/FLAIR images, which were co-registered with the T1c in the BrainLab Elements software suite, placing all segmentations in the patient T1c space. The segmentation protocol and rater performance for this imaging set are discussed in more detail in Visser et al. (2019).

**FIGURE 1:**
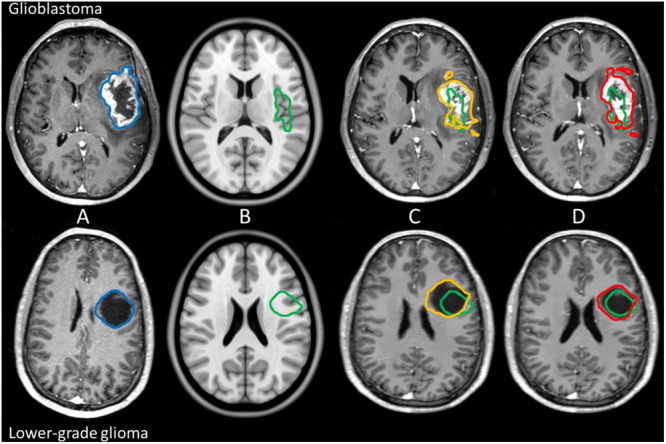
Example of manual tumor annotations followed by pre-operative linear and non-linear registration for glioblastoma and lower-grade glioma in the left hemisphere. Manual segmentation of tumor in patient space is shown in blue, the corresponding estimated target volume in anatomical reference space in green, the tumor segmentation after linear transformation in orange, and the tumor segmentation after non-linear transformation in red. The top row shows imaging of a glioblastoma patient, and the bottom row shows imaging of a lower-grade glioma patient. From left to right: patient space **(A)**, anatomical reference space **(B)**, patient space linearly transformed to anatomical reference space **(C)**, and patient space non-linearly transformed to anatomical reference space **(D)**.

**FIGURE 2:**
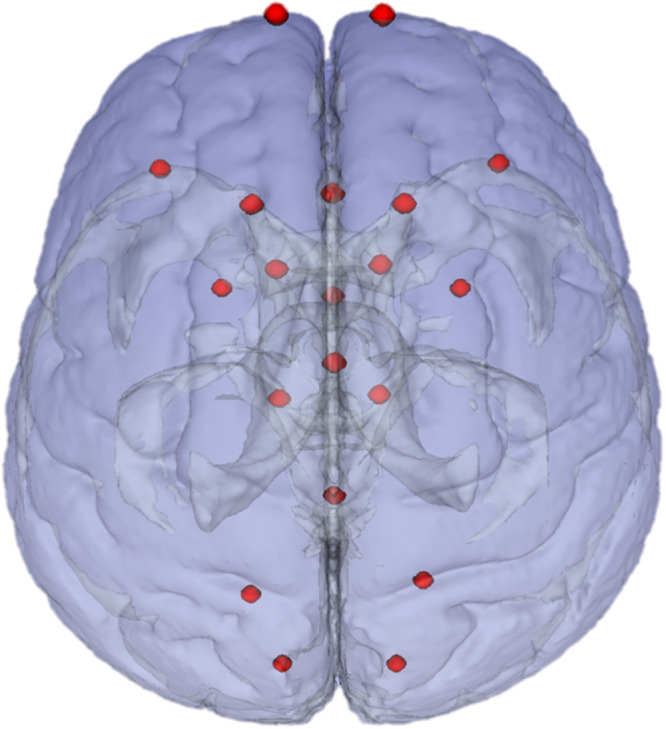
Landmarks in ICBM2009a space. Chosen landmarks are a subset of 20 chosen from the landmarks recommended in Chollet et al. (2014).

To measure tumor registration accuracy a tumor target volume in ICBM2009a space was defined, which corresponds with the tumor volume in patient space. The same rater who performed tumor segmentations in patient space also defined the tumor target volumes in ICBM2009a space. This was done by identifying the healthy tissue structures and salient edges surrounding the tumor and finding the analogous areas in the ICBM2009a template. For each segmentation in patient space a corresponding target volume of healthy tissue was delineated in MNI as ground truth for optimal registration, see Figures 1A,B. Furthermore, anatomical landmarks were placed in patient space and in anatomical reference space. A set of landmarks and their corresponding intra-rater agreement was described in Chollet et al. (2014). From these landmarks, we selected a subset of 20 landmarks based on the coverage of the supratentorial compartment and low intra-rater variation (≤2 mm), shown in Figure 2. Specific locations of the landmarks are given in Supplementary Table S1. Segmentations of target volumes in ICBM2009a space and landmark placements were performed in ITK-SNAP (Yushkevich et al., 2016).

To be able to review the intra-rater consistency, the same rater delineated the tumor target volumes again in three randomly selected enhancing and three non-enhancing gliomas on both sessions. This was done a year after the initial delineation and the rater was blinded to the initial results.

### Registration Packages

We selected registration packages from Klein et al. (2009) that are most commonly used and work with the NIfTI file format, and added two more recently developed packages: FSL (FLIRT and FNIRT) (FSL, RRID:SCR_002823), SPM5 (SPM, RRID:SCR_007037), DARTEL (SPM, RRID:SCR_007037), ANTs (SyN) (ANTS - Advanced Normalization ToolS, RRID:SCR_004757), Elastix (elastix, RRID:SCR_009619), and NiftyReg (NiftyReg, RRID:SCR_006593). For SPM related routines SPM5 indicates the registration method first introduced in SPM5, and SPM12 is the implementation we used.

### Preprocessing

DICOM data of the T1c images were converted to NIfTI with the dcm2niix tool (Li et al., 2016). Standard pre-processing was used for each method. Therefore, the pre-processing was different for FSL, ANTs, Elastix, and NiftyReg than for SPM5 and DARTEL.

#### FSL, ANTs, Elastix, and NiftyReg

T1c images were pre-processed with N4-bias correction to correct for low-frequent intensity non-uniformity (Tustison et al., 2010), anisotropic diffusion smoothing to remove noise while respecting the edges in the images (Perona and Malik, 1990), and a brain extraction routine from the ANTs image processing toolbox to obtain a brain mask in patient space (Avants et al., 2009, 2011). Of note, the brain mask in patient space was the union of healthy tissue and tumor and/or surgical cavity. A brain mask for the anatomical atlas was provided with the atlas^1^.

#### SPM5 and DARTEL

SPM5 and DARTEL registrations use the Unified segmentation approach that incorporates similar operations as above together with tissue segmentation (Ashburner and Friston, 2005). We used the CAT12 implementation in SPM12 (Wellcome Centre for Human Neuroimaging, London, United Kingdom) that has its own bias-field correction, non-local means noise filtering (Manjón et al., 2010), and brain masking (Gaser, 2009). Then T1c images were segmented to gray and white matter for later use during registration instead of the original T1c images.

### Registration Scheme

Pre- and post-operative MRI were processed independently to allow for comparison in registration performance. Tumor segmentations and landmarks in patient space were transformed to ICBM2009a space with linear transformations (Figure 1C), and non-linear transformations (Figure 1D). Due to differences in available options in each registration package the parameter settings per package were not designed to match those of other packages but were based on settings from previous work (van der Lijn et al., 2009; De Witt Hamer et al., 2013; Ou et al., 2014; Mutsaerts et al., 2018; Bartel et al., 2019) and are provided in the Supplementary Material. Furthermore, non-linear registrations were initialized with the linear transformations from the corresponding registration package.

#### FSL, ANTs, Elastix, and NiftyReg

**FIGURE 3:**
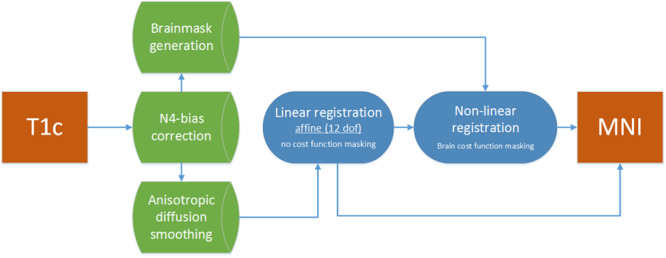
Registration flowchart. Images were bias corrected and smoothed and subsequently passed to the linear registration. Brain masks were used to drive the non-linear registration step, which was initiated with the results of the linear registration.

Linear registrations were performed without using masks in ICBM2009a space, nor in patient space. Non-linear registration was driven by the brain masks for either selecting voxels in ICBM2009a space or cost-function masking in patient space. This resulted in two registrations to ICBM2009a space for each T1c image per package: a linear and a non-linear registration. The entire registration scheme is shown in Figure 3.

#### SPM5 and DARTEL

Intermediate and final templates in the SPM format are needed for the registration to the ICBM2009a space. SPM5 and DARTEL use the ICBM152 template (Mazziotta et al., 2001), and the 1.5 mm × 1.5 mm × 1.5 mm IXI-database template (Gaser, 2009), respectively. These two templates were additionally aligned with the ICBM2009a template using geodesic shooting registration (Ashburner and Friston, 2011). The so obtained transformation was applied to all SPM5 and DARTEL results to transform them to the ICBM2009a space for comparison. Preparing the dedicated registration priors using the ICBM2009a template would provide a possible alternative approach avoiding the intermediate transformation; however, the errors introduced by the extra transformation were minimal.

We used a hybrid implementation of the DARTEL registration (Mutsaerts et al., 2018). In the region within and around the tumor we used the results of SPM5. In the surrounding tissue, the original DARTEL transformation was used. In the margin around the tumor that encompassed the maximal difference in the transformation fields on the tumor border, we have combined the SPM5 and DARTEL fields with cubic weighting depending on the distance from the lesion to ensure continuity of the merged transformation fields.

### Measures of Accuracy

#### Rater Consistency

The locations of post-operative target volumes should be consistent with their respective pre-operative target volume locations in ICBM2009a space. We define rater consistency as the percentage of post-operative voxels with a distance < 3 mm outside the pre-operative target volume. The voxels of the post-operative target volumes were assumed to be located close to the edge and preferably inside of the pre-operative target volumes, as this would be the most probable region where the surgeon might have left residual tumor.

To evaluate the intra-rater consistency on the tumor target volumes in the ICBM2009a space, the Dice score and the modified Hausdorff distance were calculated (see the following section). Additionally, the generalized conformity index (GCI = | regROI∩tarROI|/| refROIG∪tarROI|) was calculated to allow comparison with our previous study on intra-rater agreement in glioma segmentation (Visser et al., 2019).

#### Tumor Registration

The overlap between the segmentations and target volume was determined with the Dice score as:

(1) $$D\,i\,c\,e=\frac{2\,\left|r\,e\,g\,R\,O\,I\,\cap t\,a\,r\,R\,O\,I\right|}{\left|r\,e\,g\,R\,O\,I\right|+\left|t\,a\,r\,R\,O\,I\right|}$$

where |*regROI*|, |*tarROI*, and |*regROI*∩*tarROI*| are the volumes of the registered tumor segmentation, the target volume in ICBM2009a space, and their intersection, respectively. Dice scores below 0.4 were considered poor agreement, 0.4–0.6 as reasonable, 0.6–0.7 as good, and 0.7–1 as excellent (Bartko, 1991; Cicchetti, 1994).

The average distance between the borders of the registered tumor segmentation and target volume in ICBM2009a space was determined by the modified Hausdorff distance proposed for object matching in Dubuisson and Jain (1994),

(2) $$d_{\text{modH}}=\text{max}\,\left\{\frac{1}{N_{\text{R}}}\,\sum_{r\in R}d\,\left(r,T\right),\frac{1}{N_{\text{T}}}\,\sum_{t\in T}d\,\left(t,R\right)\right\}$$

where *N*_R_ and *N*_T_ are the number of vertices describing the border of the registered tumor segmentation and target volume in ICBM2009a space, respectively, *r* and *t* give a specific vertex from the border of the registered tumor segmentation and target volume in ICBM2009a space, respectively, and *d* defines the minimal distance of a point from an object, *d*(*r*,*T*) = *min*_*t* ∈ *T*_||r-t||.

#### Landmark Registration

General brain alignment accuracy was determined by an average distance in mm between the registered patient space landmarks and the corresponding landmarks in ICBM2009a space.

(3) $$d_{\text{avL}}=\frac{1}{N_{\text{L}}}\,\sum_{i=1}^{N_{\text{L}}}d\,\left(L_{\text{i}}^{\text{reg}},L_{\text{i}}^{\text{MNI}}\right)$$

where *d*_avL_ is the average distance for a single patient, *N*_L_ is the number of labels, and $d\,\left(L_{\text{i}}^{\text{reg}},L_{\text{i}}^{\text{MNI}}\right)$ is the distance between the registered landmark ($L_{\text{i}}^{\text{reg}})$ to the corresponding landmark in ICBM2009a space ($L_{\text{i}}^{\text{MNI}})$.

#### Statistical Analysis of Accuracy Measures

Linear mixed models were used to evaluate the results with Dice, *d*_modH_, or *d*_avL_ as a response variable. Type of registration package, pathology, time-point, and the type of transformation were set as fixed-effects to examine their association with accuracy results. The patient ID was set as a random effect with a random intercept, and the time-point was nested inside the patient ID. Because six registration packages were tested, the differences between all possible combinations of packages were tested with Tukey’s honestly significant difference test. A *p*-value less than 0.05 was considered significant.

## Results

### Patient Characteristics

Of the lower-grade glioma patients 12 had astrocytoma WHO grade II, four oligodendroglioma WHO grade II, three oligoastrocytoma WHO grade II, and one anaplastic astrocytoma WHO grade III (Louis et al., 2007). Of the 20 glioblastoma patients, 10 were female; median age was 65.7 years (IQR 52.1–71.1 years); and 8 patients had a tumor in the left hemisphere. Median and interquartile range for pre-operative tumor volume was 29.6 mL (IQR 10.3–55.3 mL), and for post-operative 0.8 mL (IQR 0.3–3.5 mL). Of the 20 lower-grade glioma patients, 8 were female; median age was 36.7 years (IQR 28.9–45.7); and 9 patients had a tumor in the left hemisphere. Median and interquartile range for pre-operative tumor volume was 55.2 mL (IQR 33.8–72.1 mL), and for post-operative 10.1 mL (IQR 5.5–16.9 mL).

### Rater Consistency

For glioblastoma and lower-grade glioma 73 and 87% of the voxels defined as post-operative target volume in ICBM2009a space were within 3 mm of the pre-operative target volume, respectively. The mean Dice score, the modified Hausdorff distance, and GCI for the intra-rater agreement of the delineation in the ICBM2009a space was 0.87, 1.27, and 0.77 mm for the pre-operative and 0.48, 2.21, and 0.32 mm for the post-operative enhancing gliomas; 0.87, 1.04, and 0.76 mm for the pre-operative and 0.67, 1.21, and 0.51 mm for the post-operative non-enhancing gliomas.

### Comparison of Accuracy Between Glioblastoma and Lower-Grade Glioma

**FIGURE 4:**
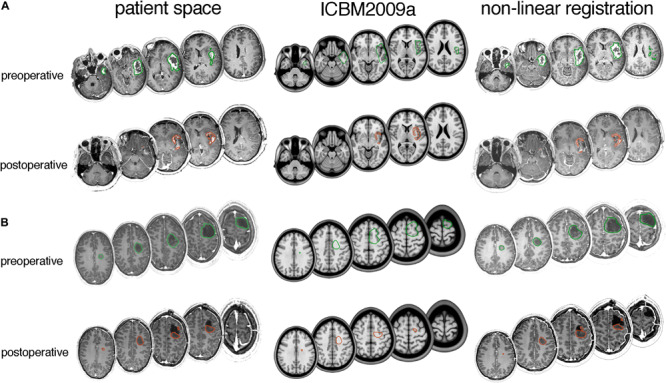
Typical tumor registration examples for **(A)** glioblastoma and **(B)** lower-grade glioma. Axial slices with delineated pre- and post-operative tumor are shown in patient space (left), ICBM2009a space (middle) and after non-linear transformation by the DARTEL registration package (right). **(A)** A 66-year-old female with a 46.4 mL preoperative tumor and 3.8 mL postoperative tumor. Dice scores are 0.67 for preoperative tumor and 0.43 for postoperative tumor, and modified Hausdorff distances are 4.1 and 2.5 mm, respectively. **(B)** A 34-year old male with a 25 mL preoperative tumor and 12 mL postoperative tumor. Dice scores are 0.62 for pre-operative tumor and 0.55 for postoperative tumor, and modified Hausdorff distances are 4.5 and 4.2 mm, respectively.

Examples of typical tumor registration accuracy are shown in Figure 4.

**FIGURE 5:**
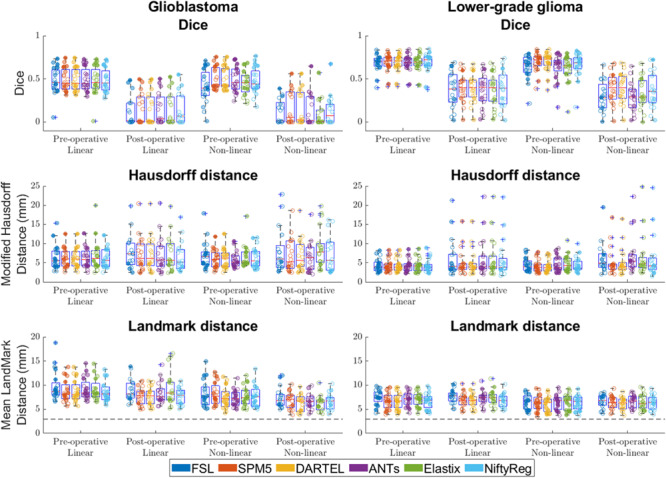
Accuracy of the six packages for both linear and non-linear registration of the pre- and post-operative time-points. Left column shows accuracy for glioblastoma, and the right column for lower-grade glioma. For Dice score higher values indicate better accuracy, for Hausdorff and mean landmark distances smaller values indicate better accuracy. The dashed line indicates the average intra-rater agreement as reported previously (Chollet et al., 2014), multiplied by two to account for error in placement in both patient and ICBM2009a space. Each dot is a data point for an accuracy measure for a single patient. Dot transparency is controlled by the volume of the tumor connected to that data point divided by the maximum tumor volume in that distribution (more transparent is smaller relative volume). The whiskers extend to the most extreme data points not considered outliers, outliers are plotted individually using the ‘+’ symbol, boxes show the interquartile range, and the contained red line shows the median of each respective distribution.

Summarizing the results from all registration packages, the median Dice scores at pre- and post-operative time-points were 0.4 and 0.0 for glioblastoma, and 0.7 and 0.3 for lower-grade glioma. The median Hausdorff distances at pre- and post-operative time-points were 5.7 and 5.8 mm for glioblastoma, and 3.8 and 4.5 mm for lower-grade glioma. The median landmark distance at pre- and post-operative time-points was 7.5 and 6.7 mm for glioblastoma, and 6.7 and 6.4 mm for lower-grade glioma. All accuracy scores are shown in Figure 5.

Compared with glioblastoma, lower-grade glioma had higher Dice scores (estimated coefficient: 0.21, 95% CI: 0.14–0.28, *p* < 0.001), lower Hausdorff distance [estimated coefficient −1.7 mm, 95% CI: (−3.3 mm–0 mm, *p* = 0.045) and lower mean landmark distance (estimated coefficient −1.2 mm, 95% CI −2.1 mm – −0.38 mm *p* = 0.006)].

### Comparison Between Pre- and Post-operative MRI Time-Points

Compared with post-operative MRI timing, pre-operative timing had higher Dice scores (estimated coefficient 0.32, 95% CI 0.26–0.39, *p* < 0.001), no difference in Hausdorff distance (estimated coefficient −1.3 mm, 95% CI −2.–0.1 mm, *p* = 0.060), and no difference in mean landmark distance (estimated coefficient 0.4 mm, 95% CI −0.3–1.2 mm, *p* = 0.259).

### Effect of Registration Software Packages

For registation accuracy differences between registration packages measured in Dice score SPM5, DARTEL, and NiftyReg did not show significant differences when compared to each other, and SPM5 and DARTEL had statistically significant higher accuracy than FSL, ANTs, and Elastix. For all significant differences the estimated coefficients were ≤0.04.

When measured with Hausdorff distance ANTs, SPM5, DARTEL, and NiftyReg did not show differences when compared to each other and SPM5 and DARTEL had statistically significant higher accuracy than FSL and Elastix. For all significant differences estimated coefficients were ≤−0.78 mm.

The registation accuracy differences between registration packages measured in landmarks for SPM5, DARTEL, and NiftyReg did not show differences when compared to each other, and had statistically significant higher accuracy than FSL, ANTs, and Elastix. For all significant differences the estimated coefficients were ≤−0.85 mm.

### Comparison of Accuracy Between Linear and Non-linear Transformation

**FIGURE 6:**
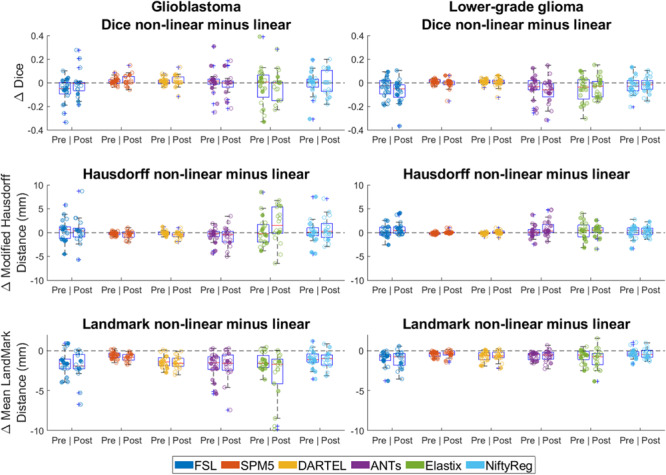
Differences between linear and non-linear registration accuracy (non-linear minus linear). Left column shows differences in accuracy for glioblastoma, and the right column for lower-grade glioma. For Dice score positive difference values indicate better non-linear performance, for Hausdorff and mean landmark distance negative difference values indicate better non-linear performance. Each dot is a data point for an accuracy measure for a single patient. Dot transparency is controlled by the volume of the tumor connected to that data point divided by the maximum tumor volume in that distribution (more transparent is smaller relative volume). The whiskers extend to the most extreme data points not considered outliers, outliers are plotted individually using the ‘+’ symbol, boxes show the interquartile range, and the contained red line shows the median of each respective distribution.

Differences between non-linear and linear registration are shown in Figure 6. Non-linear registration had lower Dice scores (effect-size −0.02, 95% CI −0.03 −−0.01 *p* < 0.001), no difference in Hausdorff distance (estimated coefficient 0.2 mm, 95% CI 0–0.4, *p* = 0.122), and lower mean landmark distance (estimated coefficient −1.1 mm, 95% CI −1.2 −−1.0, *p* = <0.001) than linear registration.

Excluding the tumor from the cost-function of the non-linear registration for FSL, ANTs, Elastix, and NiftyReg had some influence on the location and shape of the registered tumor by all registration packages, most strongly present for FSL (Supplementary Figure S1). No difference was observed in accuracy results for non-linear registration with either including or excluding the tumor from the cost-function (Supplementary Figure S2).

## Discussion

The results of this study showed that registrations with non-linear transformations were more accurate for general brain alignment, but similar to linear transformation for tumor alignment. Furthermore, registrations of lower-grade glioma were more accurate than those of glioblastoma, but registrations of pre- and post-operative MRI were similar in accuracy. SPM5 and DARTEL were slightly more accurate and FSL was slightly less accurate than the other registration packages. The manually created target volume estimates were shown to be consistent in regard to localization of pre- and post-operative tumor.

Compression of the mass effect in glioblastoma proved particularly challenging (Figure 1), which might explain why lower-grade glioma registered with higher accuracy, as these tumors typically have more infiltrative than expansive growth. Dice scores were reasonable for pre-operative glioblastoma registrations, and good for lower-grade glioma. Dice scores were poor for postoperative registration of both glioblastoma and lower-grade glioma. This was most likely a volume effect in the sense that the Dice score is more sensitive to misalignment of small volumes, since Hausdorff distances were comparable to those of pre-operative registration results (Figure 5). Upon visual inspection of the non-linear registrations, these were considered to provide a reasonable representation of the normal brain structures distant from the tumor without gross deformation. The accuracy of the tumor registrations depended on the tumor volume and extent of mass effect, which is unsurprising since regions in the patient images without analog in the reference space can – by definition – not be aligned correctly. Another source of visual registration imprecision was ventricular enlargement, which was observed in a few cases. Furthermore, unlike the source images used to create the ICBM2009a atlas, the patient images in this study were acquired after gadolinium administration, which sometimes resulted in erroneous alignment of the superior sagittal sinus with (sub)cutaneous structures at the vertex.

Although SPM5 and DARTEL often outperformed the other methods, the estimated coefficients were small. When compared to FSL the estimated coefficient was largest, and FSL was also outperformed by the other packages by a smaller margin. Therefore, these results do not indicate a preference for any of the registration packages.

An important observation here is that non-linear transformation did not improve the accuracy of tumor alignment compared to linear transformation (Figure 6). These findings indicate that non-linear transformation is not required for summaries of patient populations in probability maps of tumor segmentations. Additional advantages of omitting this step would be a reduction of processing time and avoidance of registration instability in the sense that non-linear registration is more sensitive to parameters settings and variation in input.

Lesion overlap scores for lower-grade glioma described in this study are comparable to those reported in Ou et al. (2014), who also studied ANTs and FSL. The lesion was defined as the union of the cavity and recurrent tumor. They used a similar approach, but unfortunately did not specify the tumor type and specific landmarks used. Landmark distances observed in this study are slightly higher for ANTs (∼1 mm), and lower for FSL (∼2 mm) than those reported in Ou et al. (2014), which could be a result of different landmarks used, possible difference in pathology, and difference in time-point of treatment. A similarity between our findings is that we also found lower median Hausdorff distances than median landmark distances, which is counter-intuitive. The largest landmark registration errors in this study arose from registering the frontal poles of the brain and the posterior horns of the lateral ventricles, which could be explained by possibly larger inherent differences between individuals for these structures.

Often lesions are excluded from the registration process through cost function masking to improve alignment (Brett et al., 2001), which requires the creation of lesion masks in patient space prior to registration. To justify including the tumor in the brain mask an analysis was performed for non-linear transformations that either excluded or included the tumor in the brain mask. Excluding the tumor from the registration cost function did not improve tumor overlap, nor did it improve landmark alignment. An alternative to lesion masking for improving registration performance is to perform lesion filling (Popescu et al., 2014) prior to registration.

The image data we collected was of a clinical nature, and therefore had some variety in scanner models and field strength. Previous work showed that variation in scanners and acquisition parameters leads to volumetric variation in segmentations, although registration based methods were less susceptible to these differences by correcting for differences in image geometry (Takao et al., 2011; Durand-Dubief et al., 2012). The MRI acquisition differences in this study did not appear to influence the landmark position or the tumor registration, and no systematic differences in registration accuracy were observed due to either the scanner model, or the field strength.

Recommended imaging practice for glioma patients is to obtain a T1c (Thust et al., 2018), which is often the highest resolution scan, making this the preferable scan for registration. However, T1c images are different from the T1-weighted ICBM2009a template, which does not contain contrast enhanced elements such as the blood vessels. This difference between the T1c and the ICBM2009a template adds to the challenge of successful registration of a patient scan to ICBM2009a space. Differences between patient anatomy and the ICBM2009a template may further complicate the subject-to-atlas registration. Obtaining deformation statistics of anatomical variability, and incorporating this into the registration, could be beneficial to the registration (Commowick et al., 2005).

Another challenge is the inherent difference between an image of a brain with diffuse glioma and anatomical reference space, which is often created from healthy individuals. A method for dealing with the lack correspondence between patient image and anatomical reference space is to perform tumor seeding in the anatomical reference space. This will create a simulated tumor in the anatomical reference space, making it more similar to the patient image with tumor (Dawant et al., 2002; Mohamed et al., 2006; Prastawa et al., 2009). However, the applicability of such methods depends on how well the tumor growth model mimics the actual tumor, and the required manual input that must be provided. Furthermore, the implementations of the individual tumor simulations require specific data input and are therefore not easily generalizable.

Registration accuracy might also be improved by using additional information from multiple MRI sequences or image derived information such as the image gradient. This might address, for example, the ventricular enlargement often seen in glioblastoma patients. Access to different similarity metrics for the different components or phases of the registration could also increase accuracy by allowing the user to fine-tune the registration on a step-wise basis. Furthermore, the registration accuracy can be affected by the performance of the pre-processing steps. Differences due to noise removal and bias correction are expected to be minimal between different methods. However, other pre-processing methods such as skull-stripping and identifying tissue probabilities can yield larger differences between methods and thereby have a more profound effect when used for subsequent registration schemes.

Rater consistency of segmentations in the ICMB2009a space was evaluated on a small sub-sample. It showed good agreement pre-operatively in both enhancing and non-enhancing tumors. GCI was comparable to inter-rater GCI of segmentations done by experts in patient space (Visser et al., 2019). Post-operatively, the overlap was much lower due to small tumor size, especially in enhancing tumors, and thus creating a skewed perception of reproducibility. Our earlier inter-rater study showed similar low rates of GCI in these settings (Visser et al., 2019). Moreover, modified Hausdorff distance was rather low showing good intra-rater consistency despite low GCI and Dice scores of these small ROIs. Overall, Dice scores were much higher for intra-rater consistency than when comparing the manual and automatic segmentations, and the modified Hausdorff distance was several times lower. That and the fact that we have compared the registration methods on a relative scale with the same ground-truth lead us to the conclusion that the quality of segmentations in the ICMB2009a space did not have a negative impact on the results.

Defining a framework for registration success is a complex task, due to the lack of a ground truth. Extensive manual annotations provide the most rigorous framework (Rohlfing, 2012), but come at the cost of manual labor. Overlap or distance measures alone do not provide a comprehensive registration comparison, especially when high-degrees-of-freedom algorithms are used. Therefore, we used the combination of Dice overlap, Hausdorff distance, and landmark distance to quantify our registration results. Labeling and using specific tumor regions might offer an even more detailed evaluation of the registration. However, this would require more complex manual labeling of the tumor structure and is out of the scope of the current work. Furthermore, our approach is intrinsically different than approaches that involve registrations with the ultimate aim of tumor segmentation such as Prastawa et al. (2004); Stefanescu et al. (2004), and Parisot et al. (2012), where performance can be tested with only a tumor segmentation in patient space. Agreement between raters for tumor segmentations in patient space was determined in Visser et al. (2019), but the rater agreement for the estimated target volume creation in ICBM2009a space is unknown. In this study a single rater created all target volume estimates, for which we showed that the rater was quite consistent in the creation of pre- and post-operative target volumes in ICBM2009a space. Perhaps using segmentations of multiple raters might yield more generalizable results, although this would significantly increase the workload of the study.

Accurate normalization to anatomical reference space is not only relevant to the study of glioma, but also to fMRI studies, and the study of other pathologies such as stroke which faces similar challenges in registration as glioma (Crinion et al., 2007). To incorporate registration uncertainty into summary maps of a glioma patient population, the Hausdorff accuracy results reported here can be used as an indication of the amount of regularization needed. The uncertainty in registration accuracy found here is not trivial since median Hausdorff distances range between 3.8 and 5.8 mm. The smoothing of small tumor objects, particularly surgical residue, will increase the required amount of data to perform reliable statistics.

## Conclusion

Linear transformation suffices to summarize glioma locations in anatomical reference space. The obtained summary maps should be regularized to account for a registration uncertainty of 3.8–5.8 mm. Registration is more accurate for lower-grade glioma than for glioblastoma. Pre- and post-operative MR scans were similarly accurate. No single registration software package stands out.

## Data Availability Statement

Registration settings are provided in the Supplementary Material of this manuscript. The image data used in this study is available at https://doi.org/10.17026/dans-zg9-nhrj (Hamer, 2019).

## Ethics Statement

The institutional review board at the VU University Medical Center Amsterdam approved this study (2014.336). All patients provided written informed consent for use of their clinical data for medical research. The MRI data was retrospectively collected from the hospital’s Picture Archiving and Communication System. All images were analyzed after anonymization in accordance with the General Data Protection Regulation.

## Author Contributions

MV wrote the manuscript, edited the parameter settings for FSL, ANTs, Elastix, and NiftyReg, processed the data, analyzed the data, and plotted the figures. JP edited the parameter settings for SPM5 and DARTEL, analyzed the data, and revised the manuscript. DM provided additional cavity segmentations, analyzed the data, and revised the manuscript. RE managed the digital framework, analyzed the data, and revised the manuscript. EH provided additional cavity segmentations for cost-function masking and revised the manuscript. MW, FB, MH, HM, and HV analyzed the data and revised the manuscript. JM initially supervised the study and revised the manuscript. PD provided the tumor segmentations, tumor targets and landmarks, designed the statistical analysis, analyzed the data, and revised the manuscript.

## Conflict of Interest

FB reports other from Neurology, Brain, Radiology, MSJ, and Neuroradiology, personal fees from Springer, Biogen, Roche, Apitope Ltd., IXICO Ltd., and Novartis, personal fees and other from Bayer and GeNeuro, grants from Novartis, TEVA, Merck, Biogen, IMIEU, GE Healthcare, MS Society UK, Dutch MS Research Foundation, NOW, and NIHR, outside the submitted work.

The remaining authors declare that the research was conducted in the absence of any commercial or financial relationships that could be construed as a potential conflict of interest.

## Footnotes

1 http://www.bic.mni.mcgill.ca/ServicesAtlases/ICBM152NLin2009

## References

[B1] Ashburner J. Friston K. J. (2005). Unified segmentation. *Neuroimage* 26 839–851. 10.1016/j.neuroimage.2005.02.018 15955494

[B2] Ashburner J. Friston K. J. (2011). Diffeomorphic registration using geodesic shooting and Gauss-Newton optimisation. *Neuroimage* 55 954–967. 10.1016/j.neuroimage.2010.12.049 21216294 PMC3221052

[B3] Avants B. B. Tustison N. J. Song G. Gee J. C. (2009). ANTS: open-source tools for normalization and neuroanatomy. *IEEE Trans. Med. Imaging* 10 1–11.

[B4] Avants B. B. Tustison N. J. Wu J. Cook P. A. Gee J. C. (2011). An open source multivariate framework for N-tissue segmentation with evaluation on public data. *Neuroinformatics* 9 381–400. 10.1007/s12021-011-9109-y 21373993 PMC3297199

[B5] Bartel F. Visser M. de Ruiter M. Belderbos J. Barkhof F. Vrenken H. (2019). Non-linear registration improves statistical power to detect hippocampal atrophy in aging and dementia. *NeuroImage Clin.* 23:101902. 10.1016/j.nicl.2019.101902 31233953 PMC6595082

[B6] Bartko J. J. (1991). Measurement and reliability: statistical thinking considerations. *Schizophr. Bull.* 17 483–489. 10.1093/schbul/17.3.483 1947873

[B7] Brett M. Leff A. P. Rorden C. Ashburner J. (2001). Spatial normalization of brain images with focal lesions using cost function masking. *Neuroimage* 14 486–500. 10.1006/nimg.2001.0845 11467921

[B8] Caviness V. S. Meyer J. Makris N. Kennedy D. N. (1996). MRI-based topographic parcellation of human neocortex: an anatomically specified method with estimate of reliability. *J. Cogn. Neurosci.* 8 566–587. 10.1162/jocn.1996.8.6.566 23961985

[B9] Chollet M. B. Aldridge K. Pangborn N. Weinberg S. M. DeLeon V. B. (2014). Landmarking the brain for geometric morphometric analysis: an error study. *PLoS One* 9:e86005. 10.1371/journal.pone.0086005 24489689 PMC3904856

[B10] Cicchetti D. V. (1994). Guidelines, criteria, and rules of thumb for evaluating normed and standardized assessment instruments in psychology. *Psychol. Assess.* 6 284–290. 10.1037/1040-3590.6.4.284

[B11] Commowick O. Stefanescu R. Fillard P. Arsigny V. Ayache N. Pennec X. (2005). “Incorporating statistical measures of anatomical variability in atlas-to-subject registration for conformal brain radiotherapy,” in *Medical Image Computing and Computer-Assisted Intervention – MICCAI 2005. MICCAI 2005. Lecture Notes in Computer Science (including subseries Lecture Notes in Artificial Intelligence and Lecture Notes in Bioinformatics)*, eds Duncan J. S. Gerig G. (Berlin: Springer), 927–934. 10.1007/11566489_114 16686049

[B12] Crinion J. Ashburner J. Leff A. Brett M. Price C. Friston K. (2007). Spatial normalization of lesioned brains: performance evaluation and impact on fMRI analyses. *Neuroimage* 37 866–875. 10.1016/j.neuroimage.2007.04.065 17616402 PMC3223520

[B13] Dawant B. M. Hartmann S. L. Pan S. Gadamsetty S. (2002). Brain atlas deformation in the presence of small and large space-occupying tumors. *Comput. Aided Surg.* 7 1–10. 10.1002/igs.10029 12173876

[B14] De Witt Hamer P. C. Hendriks E. J. Mandonnet E. Barkhof F. Zwinderman A. H. Duffau H. (2013). Resection probability maps for quality assessment of Glioma surgery without brain location bias. *PLoS One* 8:e73353. 10.1371/journal.pone.0073353 24039922 PMC3765204

[B15] Dubuisson M. Jain A. K. (1994). “A modified hausdorff distance for object matching,” in *Proceedings of the International Conference on Pattern Recognition*, Jerusalem, 566–568. 10.1109/ICPR.1994.576361

[B16] Durand-Dubief F. Belaroussi B. Armspach J. P. Dufour M. Roggerone S. Vukusic S. (2012). Reliability of longitudinal brain volume loss measurements between 2 sites in patients with multiple sclerosis: comparison of 7 quantification techniques. *Am. J. Neuroradiol.* 33 1918–1924. 10.3174/ajnr.A3107 22790248 PMC7964600

[B17] Ellingson B. M. Cloughesy T. F. Pope W. B. Zaw T. M. Phillips H. Lalezari S. (2012). Anatomic localization of O6-methylguanine DNA methyltransferase (MGMT) promoter methylated and unmethylated tumors: a radiographic study in 358 de novo human glioblastomas. *Neuroimage* 59 908–916. 10.1016/j.neuroimage.2011.09.076 22001163

[B18] Ellingson B. M. Lai A. Harris R. J. Selfridge J. M. Yong W. H. Das K. (2013). Probabilistic radiographic atlas of glioblastoma phenotypes. *Am. J. Neuroradiol.* 34 533–540. 10.3174/ajnr.A3253 22997168 PMC7964888

[B19] Fonov V. Evans A. McKinstry R. Almli C. Collins D. (2009). Unbiased nonlinear average age-appropriate brain templates from birth to adulthood. *Neuroimage* 47:S102 10.1016/S1053-8119(09)70884-5

[B20] Fonov V. Evans A. C. Botteron K. Almli C. R. McKinstry R. C. Collins D. L. (2011). Unbiased average age-appropriate atlases for pediatric studies. *Neuroimage* 54 313–327. 10.1016/j.neuroimage.2010.07.033 20656036 PMC2962759

[B21] Gaser C. (2009). Partial volume segmentation with adaptive maximum a posteriori (MAP) approach. *Neuroimage* 47:S121 10.1016/S1053-8119(09)71151-6

[B52] Hamer W. (2019). *PICTURE Project: Expert Manual Glioma Segmentations on MRI*. The Hague: DANS 10.17026/dans-zg9-nhrj

[B22] Hendriks E. J. Habets E. J. J. Taphoorn M. J. B. Douw L. Zwinderman A. H. Vandertop W. P. (2018). Linking late cognitive outcome with glioma surgery location using resection cavity maps. *Hum. Brain Mapp.* 39 2064–2074. 10.1002/hbm.23986 29380489 PMC5947547

[B23] Klein A. Andersson J. Ardekani B. A. Ashburner J. Avants B. Chiang M.-C. (2009). Evaluation of 14 nonlinear deformation algorithms applied to human brain MRI registration. *Neuroimage* 46 786–802. 10.1016/j.neuroimage.2008.12.037 19195496 PMC2747506

[B24] Li X. Morgan P. S. Ashburner J. Smith J. Rorden C. (2016). The first step for neuroimaging data analysis: DICOM to NIfTI conversion. *J. Neurosci. Methods* 264 47–56. 10.1016/j.jneumeth.2016.03.001 26945974

[B25] Liu T. T. Achrol A. S. Mitchell L. A. Du W. A. Loya J. J. Rodriguez S. A. (2016). Computational identification of tumor anatomic location associated with survival in 2 large cohorts of human primary glioblastomas. *Am. J. Neuroradiol.* 37 621–628. 10.3174/ajnr.A4631 26744442 PMC4833648

[B26] Louis D. N. Ohgaki H. Wiestler O. D. Cavenee W. K. Burger P. C. Jouvet A. (2007). The 2007 WHO classification of tumours of the central nervous system. *Acta Neuropathol.* 114 97–109. 10.1007/s00401-007-0243-4 17618441 PMC1929165

[B27] Manjón J. V. Coupé P. Martí-Bonmatí L. Collins D. L. Robles M. (2010). Adaptive non-local means denoising of MR images with spatially varying noise levels. *J. Magn. Reson. Imaging* 31 192–203. 10.1002/jmri.22003 20027588

[B28] Mazziotta J. Toga A. Evans A. Fox P. Lancaster J. Zilles K. (2001). A probabilistic atlas and reference system for the human brain: international consortium for brain mapping (ICBM). *Philos. Trans. R. Soc. Lond. Ser. B Biol. Sci.* 356 1293–1322. 10.1098/rstb.2001.0915 11545704 PMC1088516

[B29] Mohamed A. Zacharaki E. I. Shen D. Davatzikos C. (2006). Deformable registration of brain tumor images via a statistical model of tumor-induced deformation. *Med. Image Anal.* 10 752–763. 10.1016/j.media.2006.06.005 16860588

[B30] Müller D. M. J. Robe P. A. J. T. Eijgelaar R. S. Witte M. G. Visser M. de Munck J. C. (2019). Comparing glioblastoma surgery decisions between teams using brain maps of tumor locations, biopsies, and resections. *JCO Clin. Cancer Inform.* 3 1–12. 10.1200/CCI.18.00089 30673344 PMC6873995

[B31] Mutsaerts H. J. M. M. Petr J. Thomas D. L. De Vita E. Cash D. M. van Osch M. J. P. (2018). Comparison of arterial spin labeling registration strategies in the multi-center GENetic frontotemporal dementia initiative (GENFI). *J. Magn. Reson. Imaging* 47 131–140. 10.1002/jmri.25751 28480617 PMC6485386

[B32] Ou Y. Akbari H. Bilello M. Da X. Davatzikos C. (2014). Comparative evaluation of registration algorithms in different brain databases with varying difficulty: results and insights. *IEEE Trans. Med. Imaging* 33 2039–2065. 10.1109/TMI.2014.2330355 24951685 PMC4371548

[B33] Parisot S. Duffau H. Chemouny S. Paragios N. (2012). “Joint tumor segmentation and dense deformable registration of brain MR images,” in *Medical Image Computing and Computer-Assisted Intervention – MICCAI 2012*, eds Ayache N. Delingette H. Golland P. Mori K. (Berlin: Springer), 651–658. 10.1007/978-3-642-33418-4_80 23286104

[B34] Perona P. Malik J. (1990). Scale-space and edge detection using anisotropic diffusion. *IEEE Trans. Pattern Anal. Mach. Intell.* 12 629–639. 10.1109/34.56205

[B35] Popescu V. Ran N. C. G. Barkhof F. Chard D. T. Wheeler-Kingshott C. A. Vrenken H. (2014). Accurate GM atrophy quantification in MS using lesion-filling with co-registered 2D lesion masks. *Neuroimage Clin.* 4 366–373. 10.1016/j.nicl.2014.01.004 24567908 PMC3930097

[B36] Prastawa M. Bullitt E. Gerig G. (2009). Simulation of brain tumors in MR images for evaluation of segmentation efficacy. *Med. Image Anal.* 13 297–311. 10.1016/j.media.2008.11.002 19119055 PMC2660387

[B37] Prastawa M. Bullitt E. Ho S. Gerig G. (2004). A brain tumor segmentation framework based on outlier detection. *Med. Image Anal.* 8 275–283. 10.1016/j.media.2004.06.007 15450222

[B38] Ripollés P. Marco-Pallarés J. de Diego-Balaguer R. Miró J. Falip M. Juncadella M. (2012). Analysis of automated methods for spatial normalization of lesioned brains. *Neuroimage* 60 1296–1306. 10.1016/j.neuroimage.2012.01.094 22305954

[B39] Rohlfing T. (2012). Image similarity and tissue overlaps as surrogates for image registration accuracy: widely used but unreliable. *IEEE Trans. Med. Imaging* 31 153–163. 10.1109/TMI.2011.2163944 21827972 PMC3274625

[B40] Scott J. N. Brasher P. M. A. Sevick R. J. Rewcastle N. B. Forsyth P. A. (2002). How often are nonenhancing supratentorial gliomas malignant? A population study. *Neurology* 59 947–949. 10.1212/WNL.59.6.947 12297589

[B41] Shattuck D. W. Mirza M. Adisetiyo V. Hojatkashani C. Salamon G. Narr K. L. (2008). Construction of a 3D probabilistic atlas of human cortical structures. *Neuroimage* 39 1064–1080. 10.1016/j.neuroimage.2007.09.031 18037310 PMC2757616

[B42] Steed T. C. Treiber J. M. Patel K. Ramakrishnan V. Merk A. Smith A. R. (2016). Differential localization of glioblastoma subtype: implications on glioblastoma pathogenesis. *Oncotarget* 7 24899–24907. 10.18632/oncotarget.8551 27056901 PMC5041878

[B43] Stefanescu R. Commowick O. Malandain G. Bondiau P.-Y. Ayache N. Pennec X. (2004). “Non-rigid atlas to subject registration with pathologies for conformal brain radiotherapy,” in *Medical Image Computing and Computer-Assisted Intervention – MICCAI 2004. MICCAI 2004, Lecture Notes in Computer Science*, eds Barillot C. Haynor D. R. Hellier P. (Berlin: Springer), 704–711. 10.1007/978-3-540-30135-6_86

[B44] Takao H. Hayashi N. Ohtomo K. (2011). Effect of scanner in longitudinal studies of brain volume changes. *J. Magn. Reson. Imaging* 34 438–444. 10.1002/jmri.22636 21692137

[B45] Thust S. C. Heiland S. Falini A. Jäger H. R. Waldman A. D. Sundgren P. C. (2018). Glioma imaging in Europe: a survey of 220 centres and recommendations for best clinical practice. *Eur. Radiol.* 28 3306–3317. 10.1007/s00330-018-5314-5 29536240 PMC6028837

[B46] Tustison N. J. Avants B. B. Cook P. A. Zheng Y. Egan A. Yushkevich P. A. (2010). N4ITK: improved N3 Bias correction. *IEEE Trans. Med. Imaging* 29 1310–1320. 10.1109/TMI.2010.2046908 20378467 PMC3071855

[B47] van der Lijn F. de Bruijne M. Hoogendam Y. Y. Klein S. Hameeteman R. Breteler M. M. B. (2009). “Cerebellum segmentation in MRI using atlas registration and local multi-scale image descriptors,” in *Proceedings of the 6th IEEE International Symposium on Biomedical Imaging (2009): From Nano to Macro*, Boston, MA, 221–224. 10.1109/ISBI.2009.5193023

[B48] Viergever M. A. Maintz J. B. A. Klein S. Murphy K. Staring M. Pluim J. P. W. (2016). A survey of medical image registration – under review. *Med. Image Anal.* 33 140–144. 10.1016/j.media.2016.06.030 27427472

[B49] Visser M. Müller D. M. J. van Duijn R. J. M. Smits M. Verburg N. Hendriks E. J. (2019). Inter-rater agreement in glioma segmentations on longitudinal MRI. *NeuroImage Clin.* 22:101727. 10.1016/j.nicl.2019.101727 30825711 PMC6396436

[B50] West J. Fitzpatrick J. M. Wang M. Y. Dawant B. M. Maurer C. R. Kessler R. M. (1997). Comparison and evaluation of retrospective intermodality brain image registration techniques. *J. Comput. Assist. Tomogr.* 21 554–566. 9216759 10.1097/00004728-199707000-00007

[B51] Yushkevich P. A. Gao Y. Gerig G. (2016). “ITK-SNAP: an interactive tool for semi-automatic segmentation of multi-modality biomedical images,” in *Proceedings of the 38th Annual International Conference of the IEEE Engineering in Medicine and Biology Society, 2016 (EMBC)*, (Orlando, FL: IEEE), 3342–3345. 10.1109/EMBC.2016.7591443 PMC5493443 28269019

