# Supplementary material for: Accurate MR Image Registration to Anatomical Reference Space for Diffuse Glioma
Source: Front Neurosci. 2020 Jun 5;14:585. doi: 10.3389/fnins.2020.00585 (PMC7290158; doi:10.3389/fnins.2020.00585)
Supplement: Supplementary file 1 [file Data_Sheet_1.docx]

Supplementary Material

# Supplementary Data

## Registration scripts

### FSL

**# ----Affine registration of T1c to MNI ------------------------------------------------------------------------**

flirt
-in ${MOVING} -ref ${FIXED} -interp trilinear -dof 12 -cost mutualinfo -omat Affine.mat
-out "${T1c_in_MNI_Aff}"

**# ----Non-Linear registration of T1c to MNI -----------------------------------------------------------------**

fnirt
 --in=${MOVING} --ref=${FIXED} --aff="${WarpDIR}/${WARP_PREFIX}Aff.mat"
 --config="${CONFIG_FILE}" --inmask=${MOVINGMASK} --refmask=${FIXEDMASK}
 --cout="WARP.nii.gz"

**CONFIG_FILE:**
# GENERAL PARAMETERS

--subsamp=8,4,2,1

--miter=30,20,10,5

--infwhm=4,2,1,0

--reffwhm=4,2,1,0

--warpres=10,10,10

--splineorder=3

--jacrange=0.01,100

# MASKING OPTIONS

--applyrefmask=1

--applyinmask=1

# REGULARISATION

--regmod=bending_energy

--lambda=250,100,50,50

--ssqlambda=1

# ESTIMATE INTENSITY MAPPING

--estint=1,1,1,1

--intmod=global_non_linear_with_bias

--intorder=5

--biasres=50,50,50

--biaslambda=10000

### ANTs

T1ctoMNI_AFF="${affWARP_PREFIX}0GenericAffine.mat"

**# ----Affine registration of T1c to MNI ------------------------------------------------------------------------**

AntsRegistration

-d 3 -r [${FIXED},${MOVING},1] -u 1 -m MI[${FIXED},${MOVING},1,32,regular,0.25]

-t affine[0.1] -c [1000x1000x1000x50,1.e-6,10] -f 8x4x2x1 -l 1 -s 3x2x1x0 -o ${affWARP_PREFIX}

**# ----Non-Linear registration of T1c to MNI -----------------------------------------------------------------**

AntsRegistration

-d 3 -r [${T1ctoMNI_AFF}] -u 1 -m CC[${FIXED},${MOVING},1,4,regular,0.25]

-t SyN[0.1,${UFTV},0] -c [300x200x100x25,1.e-5,5] -f 8x4x2x1 -l 1 -z 1 -s 4x2x1x0"

-x [${FIXEDMASK},${MOVINGMASK}] -o ${WARP_PREFIX}

### Elastix

**# ----Affine registration of T1c to MNI ------------------------------------------------------------------------**

elastix

-m ${MOVING} -f ${FIXED} -p ${AffinePreset} -out ${LinWarpDir}

**AFFINEpreset**

(FixedInternalImagePixelType "float")

(MovingInternalImagePixelType "float")

(MovingImageDimension 3)

(UseDirectionCosines "true")

//Components

(Registration "MultiResolutionRegistration")

(FixedImagePyramid "FixedSmoothingImagePyramid")

(MovingImagePyramid "MovingSmoothingImagePyramid")

(Interpolator "BSplineInterpolator")

(Metric "AdvancedMattesMutualInformation")

(Optimizer "AdaptiveStochasticGradientDescent")

(ResampleInterpolator "FinalBSplineInterpolator")

(Resampler "DefaultResampler")

(Transform "AffineTransform")

(ErodeMask "false")

(NumberOfResolutions 4)

(ImagePyramidSchedule 8 8 8 4 4 4 2 2 2 1 1 1)

(HowToCombineTransforms "Compose")

(AutomaticTransformInitialization "true")

(AutomaticScalesEstimation "true")

(WriteTransformParametersEachIteration "false")

(ResultImageFormat "nii.gz")

(WriteResultImage "false")

(CompressResultImage "true")

(WriteResultImageAfterEachResolution "false")

(ShowExactMetricValue "false")

//Maximum number of iterations in each resolution level:

(MaximumNumberOfIterations 1000 )

//Number of grey level bins in each resolution level:

(NumberOfHistogramBins 32 )

(FixedKernelBSplineOrder 3)

(MovingKernelBSplineOrder 3)

//Number of spatial samples used to compute the mutual information in each resolution level:

(ImageSampler "RandomCoordinate")

(FixedImageBSplineInterpolationOrder 1 )

(UseRandomSampleRegion "false")

(NumberOfSpatialSamples 2000 )

(NewSamplesEveryIteration "true")

(CheckNumberOfSamples "true")

(MaximumNumberOfSamplingAttempts 10)

(BSplineInterpolationOrder 1)

(FinalBSplineInterpolationOrder 3)

(DefaultPixelValue 0)

**# ---- Non-Linear registration of T1c to MNI --------------------------------------**

elastix

-f ${FIXED} -m ${MOVING} -p ${BsplinePreset} -t0 ${LinWarpDir}/TransformParameters.0.txt

-mMask ${MOVINGMASK} -fMask ${FIXEDMASK}

-out ${NonLinWarpDir}

**BsplinePreset**

(FixedInternalImagePixelType "float")

(FixedImageDimension 3)

(MovingInternalImagePixelType "float")

(MovingImageDimension 3)

(UseDirectionCosines "true")

// Components

(Registration "MultiMetricMultiResolutionRegistration")

(FixedImagePyramid "FixedSmoothingImagePyramid")

(MovingImagePyramid "MovingSmoothingImagePyramid")

(Interpolator "BSplineInterpolator")

(Optimizer "StandardGradientDescent")

(ResampleInterpolator "FinalBSplineInterpolator")

(Resampler "DefaultResampler")

(Transform "BSplineTransform")

(Metric "AdvancedMattesMutualInformation" "TransformBendingEnergyPenalty")

(Metric0Weight 1.0)

(Metric1Weight 16.0)

// Setting specifics for the metrics

(NumberOfHistogramBins 32)

(FixedLimitRangeRatio 0.0)

(MovingLimitRangeRatio 0.0)

(FixedKernelBSplineOrder 3)

(MovingKernelBSplineOrder 3)

// Miscellenaneous components

(UseForegroundValue "true")

(ForegroundValue 1)

(ErodeMask "true")

(ErodeFixedMask "true")

(ErodeMovingMask "true")

// Pyramid settings

(NumberOfResolutions 4)

(ImagePyramidSchedule 8 8 8 4 4 4 2 2 2 1 1 1)

(FixedImagePyramidSmoothingSchedule 4 4 4 2 2 2 1 1 1 0 0 0)

(MovingImagePyramidSmoothingSchedule 4 4 4 2 2 2 1 1 1 0 0 0)

(MaximumNumberOfIterations 1000)

(FinalGridSpacingInPhysicalUnits 10.0 10.0 10.0)

(HowToCombineTransforms "Compose")

// Result format

(WriteTransformParametersEachIteration "false")

(ResultImageFormat "nii.gz")

(WriteResultImage "false")

(CompressResultImage "true")

(WriteResultImageAfterEachResolution "false")

(ShowExactMetricValue "false")

// Number of spatial samples used to compute the mutual information in each resolution level

(ImageSampler "RandomCoordinate")

(FixedImageBSplineInterpolationOrder 1 )

(UseRandomSampleRegion "true")

(SampleRegionSize 60.0 60.0 60.0)

(NumberOfSpatialSamples 4000 )

(NewSamplesEveryIteration "true")

(CheckNumberOfSamples "true")

(MaximumNumberOfSamplingAttempts 1000)

// Order of B-Spline interpolation used in each resolution level

(BSplineInterpolationOrder 3)

// Order of B-Spline interpolation for the transform

(BSplineTransformSplineOrder 3)

// Order of B-Spline interpolation used for applying the final deformation

(FinalBSplineInterpolationOrder 3)

// Default pixel value for pixels that come from outside the picture

(DefaultPixelValue 0)

// :: SP: Param_a in each resolution level. a_k = a/(A+k+1)^alpha ::

(SP_a 2000.0 )

// ::SP: Param_A in each resolution level. a_k = a/(A+k+1)^alpha ::

(SP_A 50.0 )

// :: SP: Param_alpha in each resolution level. a_k = a/(A+k+1)^alpha ::

(SP_alpha 0.6 )

(UseFastAndLowMemoryVersion "true")

### NiftyReg

**# ----Affine registration of T1c to MNI ------------------------------------------------------------------------**
reg_aladin

-ref "${FIXED}" -flo "${MOVING}" -aff "${WARP_PREFIX}Aff.txt"

**# ---- Non-Linear registration of T1c to MNI ----------------------------------------------------------------**

reg_f3d

-ref "${FIXED}" -flo "${MOVING}" -aff "${WarpDIR}/${WARP_PREFIX}Aff.txt" -sx 10.0 -vel

--lncc 4 -be $0.1 -smooR 2 -smooF 2

-rmask "${FIXEDMASK}" -fmask "${MOVINGMASK}"

-cpp "${WARP_PREFIX}Warp.nii.gz"

### DARTEL

CAT12 segmentation and normalization were modified to allow the following:

- Saving intermediate affine and SPM5 registrations;
- Accept TUMOR masks for cost function masking;
- Produce a combined DARTEL and SPM5 transformation as described in methods.

matlabbatch{1}.spm.tools.cat.estwrite.opts.tpm = {fullfile(x.SPMDIR, 'tpm', 'TPM.nii')};

matlabbatch{1}.spm.tools.cat.estwrite.extopts.registration.darteltpm = { fullfile(x.SPMDIR, 'toolbox', 'cat12', 'templates_1.50mm', 'Template_1_IXI555_MNI152.nii')};

matlabbatch{1}.spm.tools.cat.estwrite.extopts.registration.regstr = 0;

matlabbatch{1}.spm.tools.cat.estwrite.extopts.APP = 1070;

matlabbatch{1}.spm.tools.cat.estwrite.extopts.LASstr = 0.5;

matlabbatch{1}.spm.tools.cat.estwrite.extopts.gcutstr = 0.5;

matlabbatch{1}.spm.tools.cat.estwrite.extopts.vox = 1.5;

matlabbatch{1}.spm.tools.cat.estwrite.opts.biasstr = 0.5;

matlabbatch{1}.spm.tools.cat.estwrite.opts.samp = 3;

matlabbatch{1}.spm.tools.cat.estwrite.opts.biasstr = 0.5;

matlabbatch{1}.spm.tools.cat.estwrite.opts.affreg = 'mni';

# Supplementary Figures and Tables

For more information on Supplementary Material and for details on the different file types accepted, please see [here](http://home.frontiersin.org/about/author-guidelines#SupplementaryMaterial). Figures, tables, and images will be published under a Creative Commons CC-BY licence and permission must be obtained for use of copyrighted material from other sources (including re-published/adapted/modified/partial figures and images from the internet). It is the responsibility of the authors to acquire the licenses, to follow any citation instructions requested by third-party rights holders, and cover any supplementary charges.

## Supplementary Tables

| Landmark name | Landmark abbreviation |
| --- | --- |
| L/R frontal pole | fp-L / fp-R |
| L/R occipital pole | op-L /op-R |
| L/R temporal pole | tp-L / tp-R |
| L/R caudate nucleus centroid | cn-L / cn-R |
| L/R thalamus centroid | tha-L / tha-R |
| L/R lateral ventricle - anterior horn | lva-L / lva-R |
| L/R lateral ventricle - inferior horn | lvi-L / lvi-R |
| L/R lateral ventricle - posterior horn | lvp-L / lvp-R |
| corpus callosum - genu anterior | ccga |
| corpus callosum - midpoint superior | ccms |
| corpus callosum - splenium posterior | ccsp |
| anterior commissure midline | ac |

Supplementary Table 1: Selected landmarks for general brain alignment. Landmarks present in both hemispheres are indicated with L(eft)/R(ight). A total of 20 landmarks were selected.

## Supplementary Figures


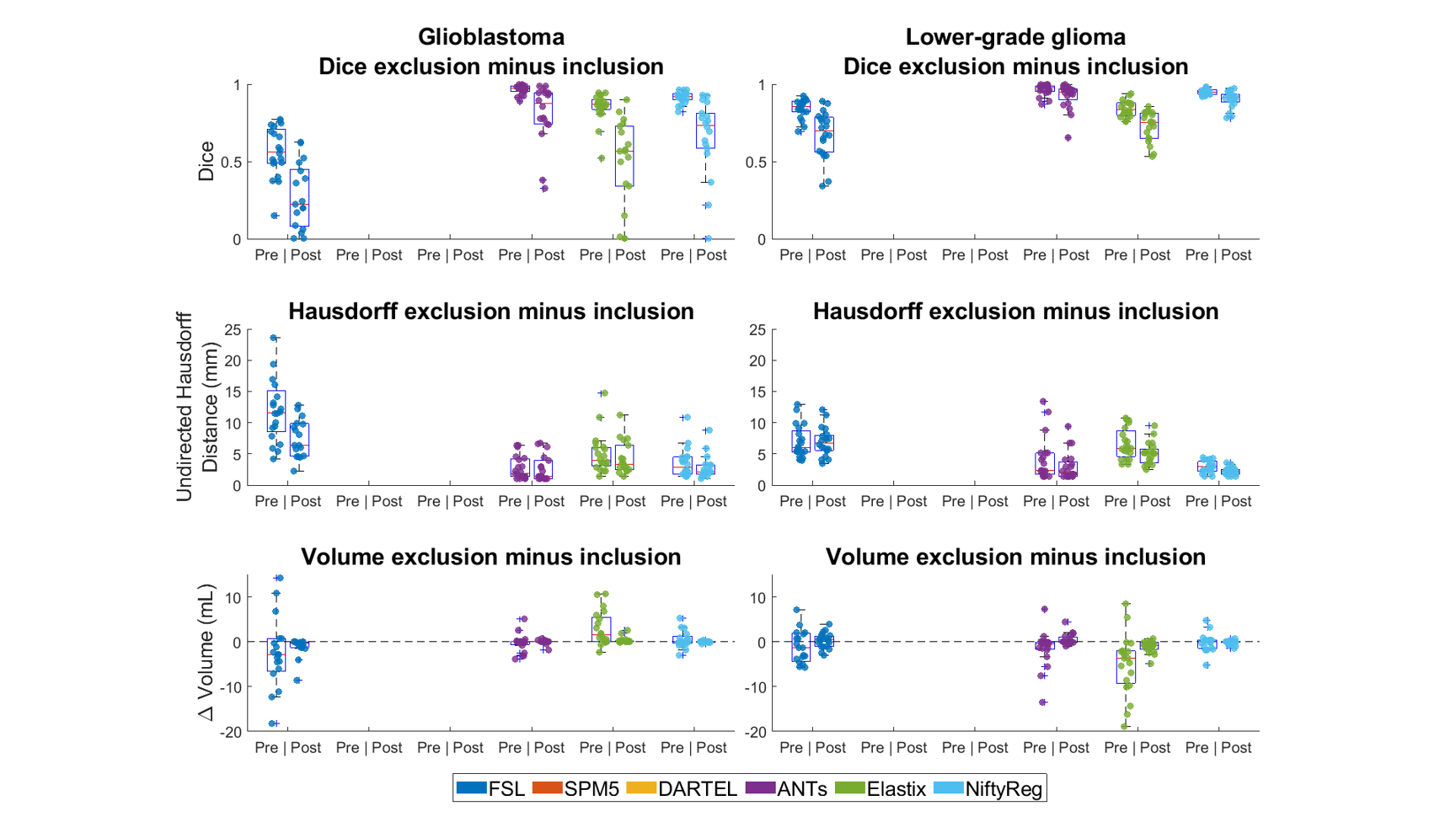
**Supplementary Figure 1:** Difference between non-linear registrations that either include or exclude the tumor from the registration cost-function. High Dice scores show little influence for including/excluding the tumor in the registration cost-function, small undirected Hausdorff distances indicate only slight deviations of the segmentation surfaces, and volume differences show changes in tumor volume due to different non-linear registration. The whiskers extend to the most extreme data points not considered outliers, outliers are plotted individually using the '+' symbol, boxes show the interquartile range, and the contained red line shows the median of each respective distribution.


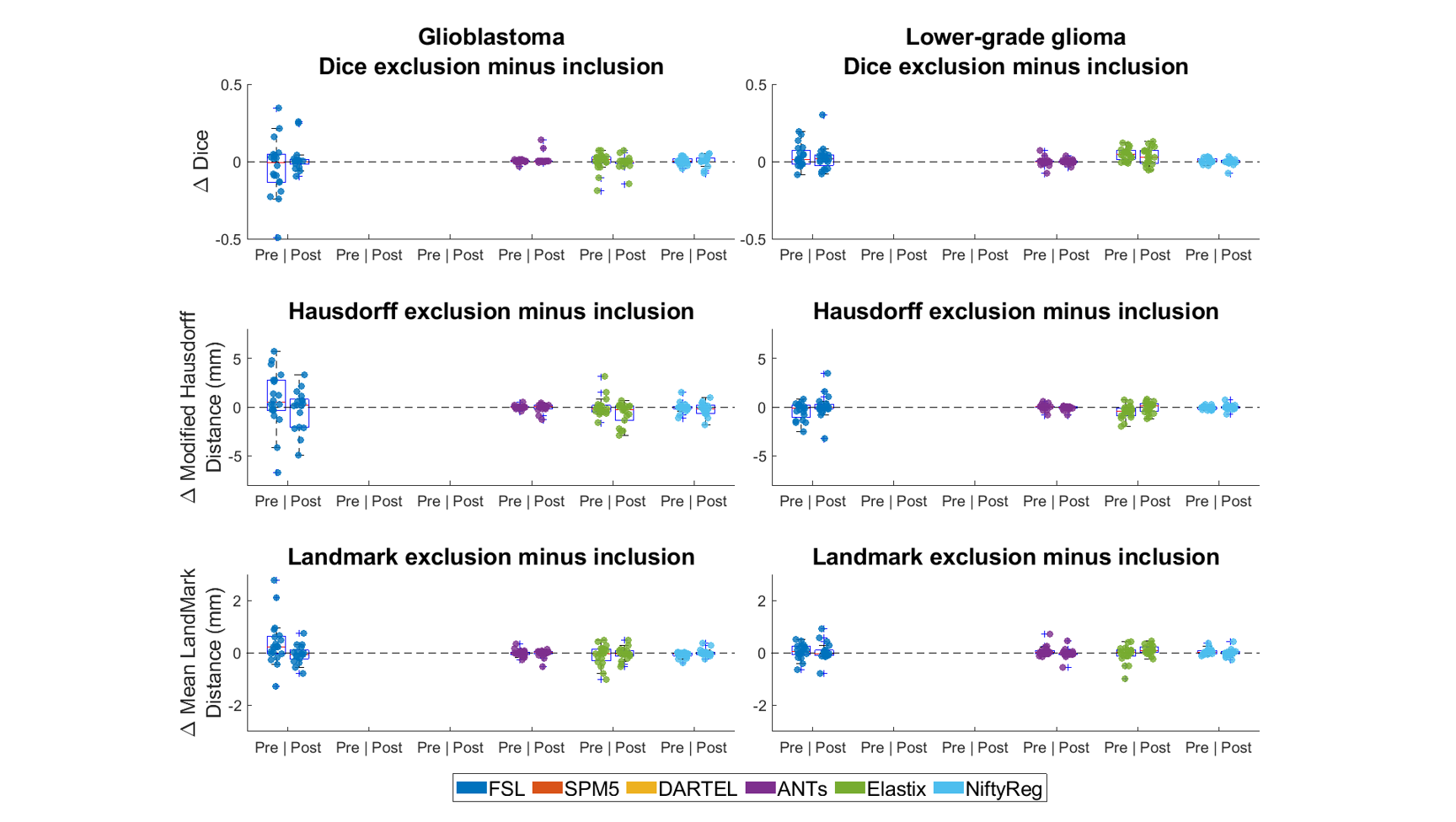


Supplementary Figure 2: Differences in registration accuracy by overlap with tumor targets and anatomical landmarks for non-linear registrations that either include or exclude the tumor from the cost-function (excluded minus included). Left column shows differences in accuracy for glioblastoma, and the right column for lower-grade glioma. For Dice scores positive difference values indicate better performance for excluded tumor, for Hausdorff and mean landmark distance negative difference values indicate better performance for excluded tumor. Each dot is a data point for an accuracy measure for a single patient. The whiskers extend to the most extreme data points not considered outliers, outliers are plotted individually using the '+' symbol, boxes show the interquartile range, and the contained red line shows the median of each respective distribution.
